# Effects of different types of mind–body exercises on depressive symptoms in older adults: a systematic review and network meta-analysis

**DOI:** 10.3389/fpubh.2026.1888299

**Published:** 2026-08-31

**Authors:** Zaiwen Wang, Ying Zhu, Wenqian Li, Pengfei Wang, Yue Liu, Yu Yin, Chengjun Li, Qian Qin, Hongyu Wang

**Affiliations:** 1 Guangxi Science and Technology Normal University, Guangxi, China; 2 College of Physical Education and Health, Guangxi Normal University, Guilin, China; 3 College of Design, Guangxi Normal University, Guilin, China

**Keywords:** depressive symptoms, mind-body exercises, older adults, systematic review, network meta-analysis

## Abstract

**Background:**

Depressive symptoms in older adults (≥60 years) have a global prevalence of 10–20%, linked to physiological decline, neurotransmitter imbalances and other factors, burdening families and healthcare resources. Existing treatments (pharmacological and non-pharmacological) have limitations, while mind–body exercises hold intervention potential due to their “mind–body linkage” trait—yet comparative effectiveness studies across different types are lacking.

**Methods:**

Systematic searches were conducted across four databases (PubMed, Cochrane Library, Embase, Web of Science) for randomized controlled trials (RCTs) on mind–body exercises for depressive symptoms in older adults. The search spanned from database inception to December 30, 2025. Two researchers independently screened studies, extracted data, and assessed quality using the Cochrane Risk of Bias Tool 2 (RoB2). Frequentist network meta-analysis (Stata 15.1) synthesized direct and indirect evidence, and the Surface Under the Cumulative Ranking Curve (SUCRA) ranked interventions by efficacy probability.

**Results:**

In the network meta-analysis of 23 randomized controlled trials, SUCRA rankings were: Tai Chi (83.3%), Pilates (71.0%), Yoga (66.3%), Qigong (59.4%), and Square Dancing (26.8%). However, Pilates was based on only 2 trials, and several comparisons had 95% CIs crossing the null, warranting cautious interpretation of the rankings.

**Conclusion:**

This study fills the precision evidence gap for mind–body exercise interventions in specific older adult populations. It suggests Tai Chi as the preferred non-drug intervention for depressive symptoms in older adults without cognitive impairment or other physical/mental disorders. Pilates, Yoga, and Qigong can be flexibly chosen per individual strength, flexibility, and social needs, while Square Dancing suits only low-risk individuals with mild loneliness-related depression. The findings support stratified clinical reference options / preliminary suggestions, backing mind–body exercises as a safe, suitable strategy for depression in older adults. Future research should standardize intervention parameters, include broader language databases, develop personalized plans, and conduct long-term follow-up and sensitivity analyses, and directly test proposed mechanisms.

**Systematic review registration:**

This study has been registered on PROSPERO (CRD420261289637).

## 1 Introduction

Depressive symptoms in older adults (aged≥60 years) are generally understood as a multifactorial mood disturbance associated with, rather than solely caused by, age-related physiological changes, altered neurotransmitter and hormonal regulation, psychosocial stressors (such as bereavement and empty nest syndrome), and chronic illness burden (1, 2). The core features of this condition manifest as alterations in cognitive function, impaired physical abilities, and a reconfiguration of social support networks, often coexisting with chronic diseases and declining self-care abilities, accompanied by organic manifestations (3, 4). The global prevalence of this disorder reaches 10 to 20% (5, 6), creating a vicious cycle of social functional impairment—chronic disease-induced bodily pain exacerbates depressive symptoms, which in turn further intensifies cognitive decline and social withdrawal, significantly increasing the risk of chronicity (7, 8). Late-life depression has also been associated with poorer functional ability, reduced quality of life, increased non-suicide mortality risk, and adverse outcomes in populations with chronic cardiometabolic diseases; suicide risk remains an important clinical concern in this age group (9). Additionally, the suicide completion rate associated with depression is higher than in other age groups, imposing a heavy burden on family caregiving and social healthcare resources (10). Against the backdrop of an accelerating global aging process (11), depressive symptoms in older adults have emerged as a critical public health challenge that requires urgent exploration of safe, effective, and age-appropriate intervention strategies.

The current treatment of depressive symptoms in older adults primarily encompasses pharmacological and non-pharmacological approaches. Pharmacological treatment can effectively alleviate core symptoms of moderate to severe depression, such as low mood and suicidal ideation, by regulating neurotransmitters (12). However, its application is significantly limited by physiological and clinical characteristics among older adults, including impaired liver and kidney function, slow metabolism (which may lead to drug accumulation) (13), an increased risk of drug interactions, poor tolerance to adverse effects, decreased efficacy in the presence of comorbid cognitive impairment, and suboptimal medication adherence (14, 15). Non-pharmacological therapies, centered on psychological interventions, align well with the biopsychosocial model of the complex pathogenesis of late-life depression (16) and can help mitigate risks associated with medication (15, 17). Importantly, psychological therapies should not be regarded as biologically inert; neuroimaging evidence suggests that psychotherapy can induce neuroplastic changes and modulate cortical–limbic pathways in depression (18, 19). Nevertheless, their real-world implementation among older adults may still be constrained by access, adherence, physical frailty, mobility limitations, caregiving resources, and the need for trained professionals (20).

Exercise interventions, as a safe and universally applicable non-pharmacological option, address the core challenges of depression through multiple dimensions at the intervention level. Physiologically, they promote the secretion of neurotransmitters and improve the physiological foundations related to emotional regulation (21). Psychologically, they enhance self-worth and reduce negative cognition (22). Socially, they foster social connections (23). This approach avoids the risks associated with medication while being suitable for both healthy older adults and those suffering from depression, thus offering a broader coverage. Additionally, at the preventive level, exercise helps delay physiological decline, strengthen emotional resilience, and solidify social support networks, thereby creating a comprehensive health protection barrier that fills the gap left by traditional interventions, which often emphasize treatment over prevention (22). Among various forms of exercise, mind–body exercises may be particularly relevant because they combine gentle physical movement with breathing regulation, attention, and elements of mindfulness. However, the current evidence does not justify a broad claim that mind–body exercise is superior to all other forms of exercise. Therefore, the present study focuses specifically on comparative effectiveness among different mind–body exercise types. Their low-to-moderate intensity, simple movements, limited equipment requirements, and flexibility for individual or group practice may support adherence and help optimize outcomes across physiological, psychological, and social dimensions (24–27).

Previous meta-analyses have established the overall effectiveness of mind–body exercises in improving cognitive function among older adults (24). However, there are notable disconnections between study designs and target populations. Some studies primarily focus on patients with mild cognitive impairment, concentrating on cognitive enhancement, without specifically designing intervention protocols and analyses for older adults without cognitive impairment (28). Other studies have included participants with a range of psychiatric or physical conditions, such as stroke and schizophrenia, resulting in heterogeneous study populations that complicate the isolation of unrelated confounding factors, thereby failing to provide tailored evidence for solely depressed individuals and healthy older adults (29). Moreover, there is a critical lack of comparative effectiveness analyses and hierarchical rankings of different types of mind–body exercises specifically targeting older adults who are “free of cognitive impairment and other psychiatric or physical diseases.” This gap has left the optimal intervention strategies for this population unclear. To address this research gap and provide tailored, precise evidence-based support for the target population of this study, we will conduct a systematic review and network meta-analysis of rigorously selected high-quality randomized controlled trials. The inclusion criteria excluded studies involving diagnosed cognitive impairment and severe psychiatric/neurological disorders. Studies involving stable, common age-related musculoskeletal or mobility limitations were retained when these conditions were not the primary target of the depression outcome and when relevant data were extractable. The study will have dual objectives: first, to comprehensively assess the comparative effectiveness of various mind–body exercises on depressive symptoms within this specific group, and second, to clarify the optimal intervention methods through hierarchical ranking.

## 2 Methods

### 2.1 Protocol and registration

This systematic review was conducted in strict accordance with the Preferred Reporting Items for Systematic Reviews and Meta-Analyses (PRISMA) 2020 Statement, in full compliance with its requirements for study inclusion criteria, data organization procedures, statistical analysis methods, and result reporting standards. The study protocol was prospectively registered with the International PROSPERO under the unique identification number CRD420261289637.

### 2.2 Data sources and search strategy

A comprehensive systematic search was conducted across four electronic databases (PubMed, Cochrane Library, Embase, and Web of Science) to identify literature on different mind–body exercise interventions for depression in older adults. To ensure complete coverage, the reference lists of eligible studies were screened for additional publications. The search timeframe included records from the inception of the databases up to December 30, 2025. The search strategy was developed according to the PICOS criteria (Population: older adults; Intervention: mind–body exercises; Comparison: any control; Outcome: depression indicators; Study design: randomized controlled trials). The primary search terms included: “mind–body exercise” or “Tai Chi” or “Qigong” or “Yoga” or “Pilates” and “older adults” and “depression” or “depressive disorder” and “randomized controlled trial.” Chinese databases were not included because the protocol restricted inclusion to full-text English-language publications; this decision may have led to omission of relevant studies on Tai Chi, Qigong, and Square Dancing and is acknowledged as a limitation. For detailed search strategies, please refer to Appendix B and B1.

### 2.3 Study selection

Following the execution of the aforementioned search strategy, two researchers (ZWW and HYW) independently conducted the literature screening in accordance with the PRISMA guidelines. The initial screening phase involved reviewing titles and abstracts to identify potentially eligible studies based on the predefined inclusion criteria. Studies that met the preliminary screening criteria were subsequently subjected to full-text retrieval and critical appraisal. The final selection of studies included for quantitative synthesis was reached through team discussions, achieving consensus through multiple rounds of iterative communication to resolve any discrepancies until a unified conclusion was formed.

### 2.4 Inclusion and exclusion criteria

The selection criteria for studies included in this systematic review were established based on the PICOS framework. Studies will be included if they meet all of the following criteria:

1. Study Design: Randomized controlled trials.
2. Participants: Older adults (aged ≥60 years; consistent with the definition of “older adults” provided in the introduction).
3. Interventions: Mind–body exercises (such as Tai Chi, Qigong, Yoga, Pilates, or dance-based mind–body exercise), with sufficient descriptions of intervention type, session duration, frequency, and total intervention period. Exercise intensity indicators (e.g., %HRmax, METs, or perceived exertion) were extracted when reported but were not required as a strict eligibility criterion because many older-adult exercise RCTs do not quantify intensity uniformly.
4. Outcomes: Measurement of depression severity data using validated scales before and after the intervention.
5. Data Completeness: Sufficient original or extractable data (including means, standard deviations, sample sizes, and precise *p*-values) to calculate effect sizes (e.g., standardized mean differences).
6. Language: Full-text articles published in English.

Studies will be excluded if they meet any of the following criteria:

1. Study Design: Observational studies (such as cross-sectional studies, case–control studies, cohort studies).
2. Participants: Non-older adults (aged <60 years), or individuals with diagnosed mild cognitive impairment, dementia, Parkinson’s disease, stroke, schizophrenia, or other severe neuropsychiatric disorders that could substantially confound depressive symptom assessment. Trials involving clinically stable common age-related musculoskeletal or mobility limitations (e.g., osteoarthritis or wheelchair use) were not automatically excluded if depressive symptoms were measured using validated scales and the intervention was applicable to older adults.
3. Interventions: Mind–body exercise interventions with insufficient details on type, duration, frequency, or total period, or combined interventions in which the independent effect of exercise could not be differentiated (e.g., yoga used in conjunction with dietary supplements).
4. Study Types: Qualitative studies, reviews, theses/dissertations, or conference proceedings.
5. Data Completeness: Lack of critical outcome data or non-extractable data (e.g., absence of numerical descriptive statistics).
6. Ethical Issues: Violations of ethical standards (e.g., lack of informed consent, disproportionate risk–benefit ratio).
7. Language: Non-English full-text articles. Full-text articles not published in English were excluded to ensure consistency in data extraction and interpretation and to avoid potential bias caused by cross-language translation of outcome indicators and results.

### 2.5 Data extraction

To guarantee the rigor of the literature search and screening process, two researchers (ZWW and HYW) independently reviewed titles, abstracts, and full texts post-search, with inter-rater reliability (Cohen’s kappa) computed for both screening stages—initial title and abstract review and subsequent full-text review. Consistency was classified into three grades: fair agreement (0.40–0.59), good agreement (0.60–0.74), and excellent agreement (>0.75) (30).

This systematic review formulated literature selection, inclusion and exclusion criteria based on the PICOS framework, with two researchers independently extracting data using a standardized protocol. Any discrepancies arising in the extraction process were resolved via consensus discussions among the research team. The information retrieved from each included study covered the following aspects:

1. Descriptive details: First author, publication year and country of origin;
2. Participant traits: Age range, gender distribution and sample size;
3. Intervention specifics: Temporal parameters (single session duration), frequency (weekly session count) and total intervention cycle (weeks);
4. Outcome metrics: Outcome Measures: Assessment indicators for depression severity in older adults, including validated psychological measurement scales.

For studies where intervention parameters or outcome data were only presented graphically without exact numerical values, Engauge Digitizer software (v12.1) was utilized for precise data extraction. In trials featuring multiple follow-up evaluations, only immediate post-intervention data were incorporated to guarantee temporal comparability. For studies lacking reported standard deviation (SD), this metric was calculated from the 95% confidence interval (CI) of group means using relevant formulas.

### 2.6 Quality assessment

We employed the Cochrane Risk of Bias (RoB2) assessment tool to evaluate study quality across five domains: (1) randomization process; (2) deviations from intended interventions; (3) missing outcome data; (4) measurement of outcomes, and (5)selection of reported results. Based on these criteria, each study was systematically evaluated for overall bias and classified into one of three categories: low risk, high risk, or some concerns. RoB2 was selected for its superior focus on bias direction and impact specific to RCTs, compared with the PEDro scale which only provides a quantitative quality score without assessing selective reporting bias.

### 2.7 Statistical analysis

For continuous outcome measures, the standardized mean difference (SMD) and its corresponding 95% CI were calculated in this study. SMD was used to standardize depression severity scores across different validated assessment scales, with a negative value indicating reduced depressive symptoms in the intervention group; effect sizes were interpreted per Cohen’s criteria. Statistical heterogeneity was assessed using the chi-square test *p*-value and *I^2^* statistic, where *I^2^* values > 50% were defined as moderate heterogeneity and > 75% as high heterogeneity. Given the variability in measurement scales across included studies, a random-effects model was employed to estimate pooled effect sizes, ensuring result consistency and improving comparability. To address cross-study scale heterogeneity (e.g., different measurement tools, intervention protocols), an inverse variance-weighted random-effects model was adopted, which explicitly accounts for between-study variance via τ^2^ estimation. This conservative approach retains methodological simplicity while enhancing comparative consistency. In accordance with the PRISMA-NMA guidelines, a frequentist framework was prioritized over a Bayesian approach to optimize result interpretability and avoid computational challenges associated with Markov chain Monte Carlo convergence. We considered sensitivity analyses to assess robustness, particularly by excluding studies with high overall risk of bias and by examining whether rankings were driven by sparsely connected nodes. However, because several interventions were represented by only two to four RCTs and some direct comparisons were supported by single studies, formal leave-one-out sensitivity analyses were not statistically stable for all nodes; therefore, robustness was interpreted qualitatively and the uncertainty was emphasized in the Results and Discussion.

The network meta-analysis was implemented using a frequentist method in Stata 15.1. During data preparation, an evidence network plot was constructed using the “network” package: each node represented an intervention or control condition, node size reflected the relevant sample size, and line thickness reflected the number of direct RCT comparisons. Direct and indirect evidence was synthesized within the random-effects network model. Node-splitting tests were performed for all pairwise comparisons with available direct head-to-head RCT evidence to assess local inconsistency, with complete results including network estimates, direct estimates, indirect estimates, and exact inconsistency *p*-values reported. SUCRA values were calculated to present probabilistic rankings, with higher values indicating a higher ranking probability rather than definitive clinical superiority. Potential publication bias was assessed using a funnel plot. Several pairwise comparisons exhibited extremely wide 95% CIs, which may stem from sparse-network issues due to uneven study distribution and small sample sizes across interventions; therefore, these results require cautious interpretation. The quality of evidence for all network meta-analysis estimates was assessed using the GRADE-NMA framework.

## 3 Results

### 3.1 Trial selection

To guarantee the rigor of the literature search and screening process, two researchers (ZWW and HYW) independently screened titles, abstracts, and full texts post-literature search. Inter-rater reliability (Cohen’s kappa) was evaluated across the two screening phases—title/abstract screening and full-text screening—with reviewer consistency classified into three grades: moderate (0.40–0.59), good (0.60–0.74), and excellent (>0.75).

**Figure 1:**
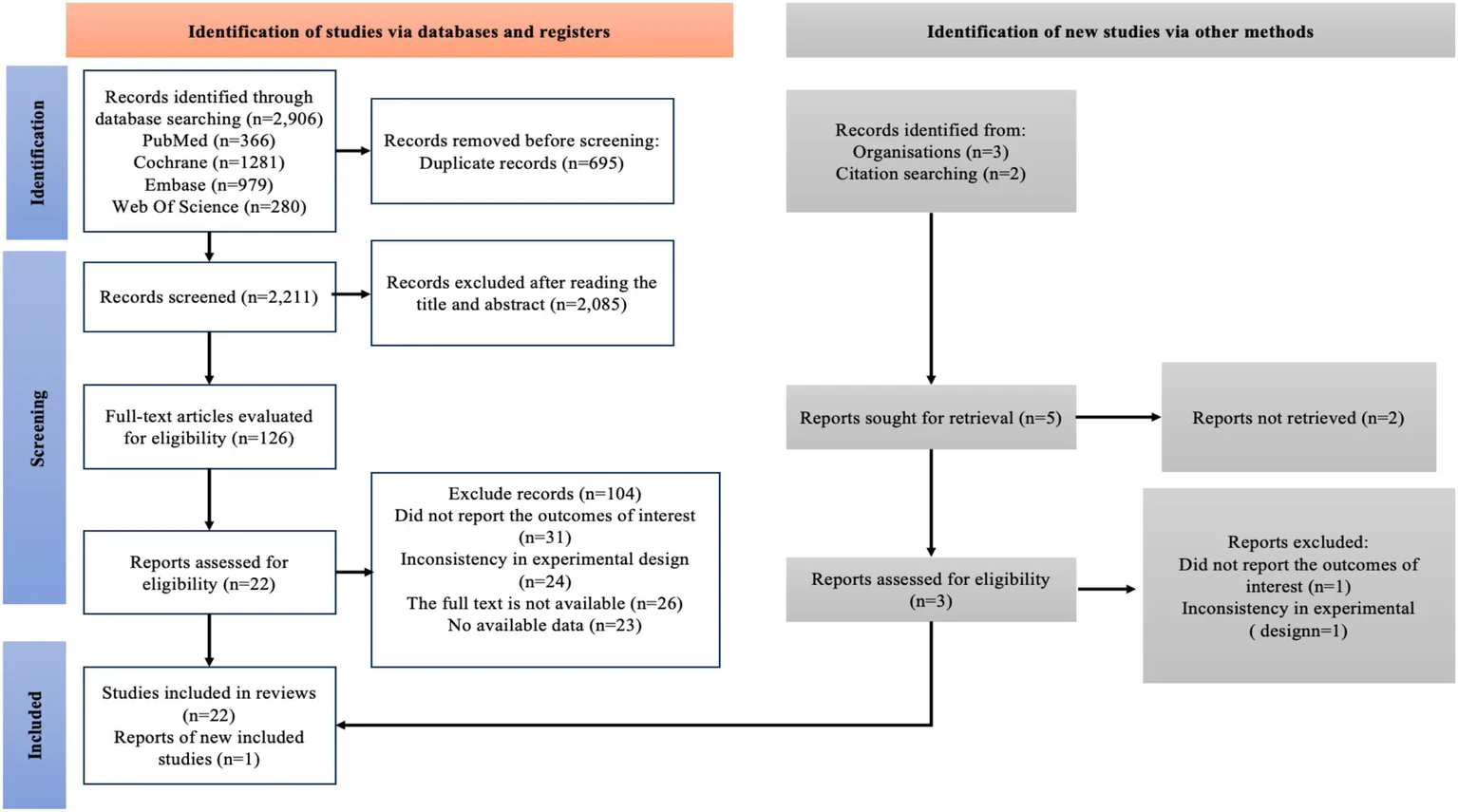
A summary of the evidence searches and selection process.

During the initial search, a comprehensive search was conducted across four electronic databases from their inception until December 30, 2025, resulting in the identification of 2,906 articles. After removing duplicates (n = 695), 2,211 articles remained for further assessment. Through title and abstract screening, 2,085 articles were excluded, leaving 126 articles for full-text review. At this point, the inter-rater reliability between the two evaluators was deemed “good” (Cohen’s kappa = 0.73). Following the full-text review, 104 articles were further excluded: 31 did not report outcomes, 24 had inconsistent experimental designs, 26 were missing full texts, and 23 lacked available data. Following full-text review, 22 studies were retained, and 1 additional eligible study was identified through reference list screening, resulting in a total of 23 included RCTs (Figure 1). At this stage, the inter-rater reliability between the two evaluators was rated as “excellent” (Cohen’s kappa = 0.81).

### 3.2 Trial characteristics

Table 1 summarizes the characteristics of the studies included in this analysis. A total of 23 articles (31–53) were incorporated, published between 2004 and 2025. Notably, China contributed the highest number of publications, with a total of six articles. The sample size of the intervention groups ranged from 7 to 67 participants, encompassing a total of 728 older adults. In the control groups, the sample size varied from 7 to 66 participants, totaling 757 older adults. In both groups, the average age exceeded 63 years.

**Table 1:** Summary table of included reviews.

Study	Country	N (IG; CG)	Age (IG; CG)	Intervention (IG)			Intervention (CG)			Participant characteristics	Outcomes
				Intervention content	Intervention time, frequency, period	Type	Intervention content	Intervention time, frequency, period	Type		
Liu et al. (31)	China	30;30	68.30 ± 4.90 68.60 ± 5.62	Tai Chi	60 min, 3 weekly 24 weeks	Tai Chi	NI	NI	NI	Older Chinese adults with depressive symptoms	GDS
Song et al. (32)	China	20; 20	67.45 ± 4.21 66.85 ± 3.53	Tai Chi	60 min, 3 weekly 12 weeks	Tai Chi	Health education and home-based exercise instructions	60 min, 3 weekly 12 weeks	RC	Older women with stable knee osteoarthritis	SDS
Hsu et al. (33)	Australia	30;30	80.73 ± 9.68 81.77 ± 6.32	Tai Chi	40 min, 3 weekly 26 weeks	Tai Chi	usual standard care	40 min, 3 weekly 26 weeks	RC	Older adults using wheelchairs	GDS
Chou et al. (34)	China	7; 7	72.6 ± 4.2 72.6 ± 4.2	Tai Chi	45 min 3 weekly 3 months	Tai Chi	NI	NI	NI	Chinese older patients with depressive disorders	CES-D
Kilpatrick et al. (35)	America	21; 19	67.1 ± 7.4 68.2 ± 5.6	Tai Chi	60 min, 1 weekly, 12 weeks	Tai Chi	Health education and home-based exercise instructions	60 min, 1 weekly, 12 weeks	RC	Older adults with geriatric depression	GDS
Lavretsky et al. (36)	American	62; 63	69.2 ± 6.9 69.4 ± 6.2	Qigong	60 min, 1 weekly, 12 weeks	Qigong	Health education and home-based exercise instructions	60 min, 1 weekly, 12 weeks	RC	Older adults with geriatric depression	HAM-D
Chan et al. (37)	China	24; 22	75.4 ± 5.9 79.4 ± 8.5	Qigong	60 min 2 weekly 3 months	Qigong	standard care control	60 min 2 weekly 3 months	RC	Hidden/community-dwelling older adults	MHI
Noradechanunt et al. (38)	Australia	13; 13	67.29 ± 8.3 65.2 ± 6.7	Tai Chi	90 min, 2 weekly, 12 weeks	Tai Chi	Health education	90 min, 2 weekly, 12 weeks	RC	Community-dwelling older adults	CES-D
Yoon et al. (39)	Korea	30; 31	60.37 ± 5.58 60.29 ± 7.72	Qigong	90 min, 1 weekly, 6 weeks	Qigong	NI	NI	NI	Older adults with depressive disorder	HAM-D
Roswiyani et al. (40)	Indonesia	67; 65	71.90 ± 8.57 74.31 ± 9.57	Qigong	90 min, 2 weekly, 8 weeks	Qigong	NI	NI	NI	Nursing-home older adults	BDI-II
Krause-Sorio et al. (41)	American	11; 11	61.5 ± 6.6 64.6 ± 6.4	Yoga	60 min 1 weekly 12 weeks	Yoga	Memory Enhancement Training	60 min 1 weekly 12 weeks	RC	Older adults with depressive disorder	BDI
Curi et al. (42)	Brazil	31; 30	64.25 ± 0.14 63.75 ± 0.08	Pilates	60 min 2 weekly 16 weeks	Pilates	NI	NI	NI	Nursing-home older adults	GHQ-12
Haboush et al. (43)	American	10; 12	69.38 ± 5.43 69.38 ± 5.43	Square dance exercise	45 min 1 weekly 8 weeks	Square dance	NI	NI	NI	Older adults with depressive disorder	GDS
Choi et al. (44)	South Korea	33; 30	77.6 ± 5.69 78.8 ± 5.83	Yoga	60 min 4 weekly 12 weeks	Yoga	NI	NI	NI	Older women	GDS
De Oliveira et al. (45)	Brazil	16; 16	63.6 ± 1.0 64.2 ± 0.8	Pilates	60 min 2 weekly 12 weeks	Pilates	home-based exercise instructions	60 min 2 weekly 12 weeks	RC	Older adults with geriatric depression	SF-36 MHS
Baklouti et al. (46)	Tunisia	65; 95	65–80 65–80	Yoga	80 min 2 weekly 8 weeks	Yoga	NI	NI	NI	Older Adults	DASS-21
Chen et al. (47)	China	62; 66	65.77 ± 4.32 72.42 ± 6.04	Yoga	70 min 3 weekly 24 weeks	Yoga	NI	NI	NI	Older Adults	TDQ
Hola et al. (48)	Czech Republic	20; 16	70.3 ± 3.8 70.3 ± 3.8	Square dance exercise	90 min 2 weekly 12 weeks	Square dance	Tai Chi	90 min 2 weekly 12 weeks	Tai Chi	Older Adults	GDS
Vankova et al. (49)	Czech Republic	42; 45	82.7 ± 6.3 82.4 ± 7.9	Square dance exercise	60 min, 1 weekly, 12 weeks	Square dance	usual standard care	60 min, 1 weekly, 12 weeks	RC	Nursing-home residents	GDS
Eyigor et al. (50)	Turkey	19; 18	73.5 ± 7.6 71.2 ± 5.5	Square dance exercise	60 min, 3 weekly, 8 weeks	Square dance	NI	NI	NI	Older women	GDS
Dos Santos et al. (51)	Brazil	9; 13	66.2 ± 4.6 73.6 ± 8.0	Square dance exercise	50 min, 2 weekly, 12 weeks	Square dance	NI	NI	NI	Community-program older women	POMS
Dong et al. (52)	Korea	32; 34	73.5 ± 5.8 75 ± 5.77	Qigong	60 min, 3 weekly, 12 weeks	Qigong	NI	NI	NI	Older women	GDS
Jing et al. (53)	China	39; 40	75.31 ± 8.79 74.72 ± 8.83	Qigong	40 min, 5 weekly, 24 weeks	Qigong	Cognitive Behavioral Therapy	40 min, 5 weekly, 24 weeks	RC	Housebound older adults	GDS

IG, Intervention group; CG, Control group; N, Number; NI, No intervention; RC, Routine care; GDS, Geriatric Depression Scale; SDS, Self-Rating Depression Scale; BDI, Beck Depression Inventory; CES-D, Center for Epide miological Studies Depression Scale; MHI, Mental Health Inventory; POMS, Profile of Mood States; SF-36 MHS, SF-36 Mental Health Summary; POMS, Profile of Mood States; DASS-21, Depression Anxiety Stress Scales-21 Items; HAM-D, Hamilton Depression Rating Scale; MHI, Mental Health Inventory; GHQ-12, General Health Questionnaire-12 Items; TDQ, Taiwanese Depression Questionnaire.

Tai Chi (7 studies): Characterized by gentle and slow movements that integrate dynamic and static elements, this practice focuses on the coordination of breath and intention, potentially exerting antidepressant effects by regulating neurotransmitter balance (31–35, 38, 48). Qigong (6 studies): Featuring simple, fluid movements aimed at harmonizing internal organs and energy flow, it can improve depressive-related pathological conditions by reducing levels of inflammatory factors (36, 37, 39, 40, 52, 53). Yoga (4 studies): Emphasizing muscle stretching and a balance of strength and flexibility, yoga works to alleviate physical discomfort and emotional lows associated with depression by enhancing meridian circulation and improving blood flow to the limbs (41, 44, 46, 47). Square Dancing (5 studies): Defined by its collective nature and strong rhythm, this activity involves simple, easy-to-learn movements set to popular music, combining aerobic exercise with social interaction, which may alleviate depressive symptoms by releasing emotions and strengthening social connections (43, 48–51). Pilates (2 studies): Focused on core muscle strengthening, body control, and breath regulation, Pilates emphasizes movement precision and mind–body coordination, assisting in the alleviation of depression among older adults by reducing bodily tension, enhancing body awareness, and improving emotional regulation skills (42, 45).

Non-physical and non-mental exercise interventions include no intervention and usual care. Common measurement tools for depression in Older Adults include the Geriatric Depression Scale (GDS), Self-Rating Depression Scale (SDS), Beck Depression Inventory (BDI), Cornell Depression Score (CDS), Center for Epidemiological Studies Depression Scale (CES-D), Hospital Anxiety and Depression Scale (HADS), Center for Epidemiological Studies Depression Scale (CES-D), Beck Depression Inventory-II (BDI-II), Profile of Mood States (POMS), etc.

### 3.3 Risk of bias

Among the 23 included studies, 19 were rated as having a low risk of bias in the randomization process, 4 did not provide specific details regarding their randomization procedures. Regarding the risk of bias in the measurement of outcomes, 22 studies were assessed as low risk, while 1study was classified as high risk. In terms of selective reporting bias, all 23 studies were deemed to be at low risk. Based on the five criteria, the overall risk of bias for the 23 studies was categorized as follows: 12 studies were identified as having a low overall risk of bias, 9 were assessed as having a high overall risk, and 2 were assessed as having “some concerns”.

**Figure 2:**
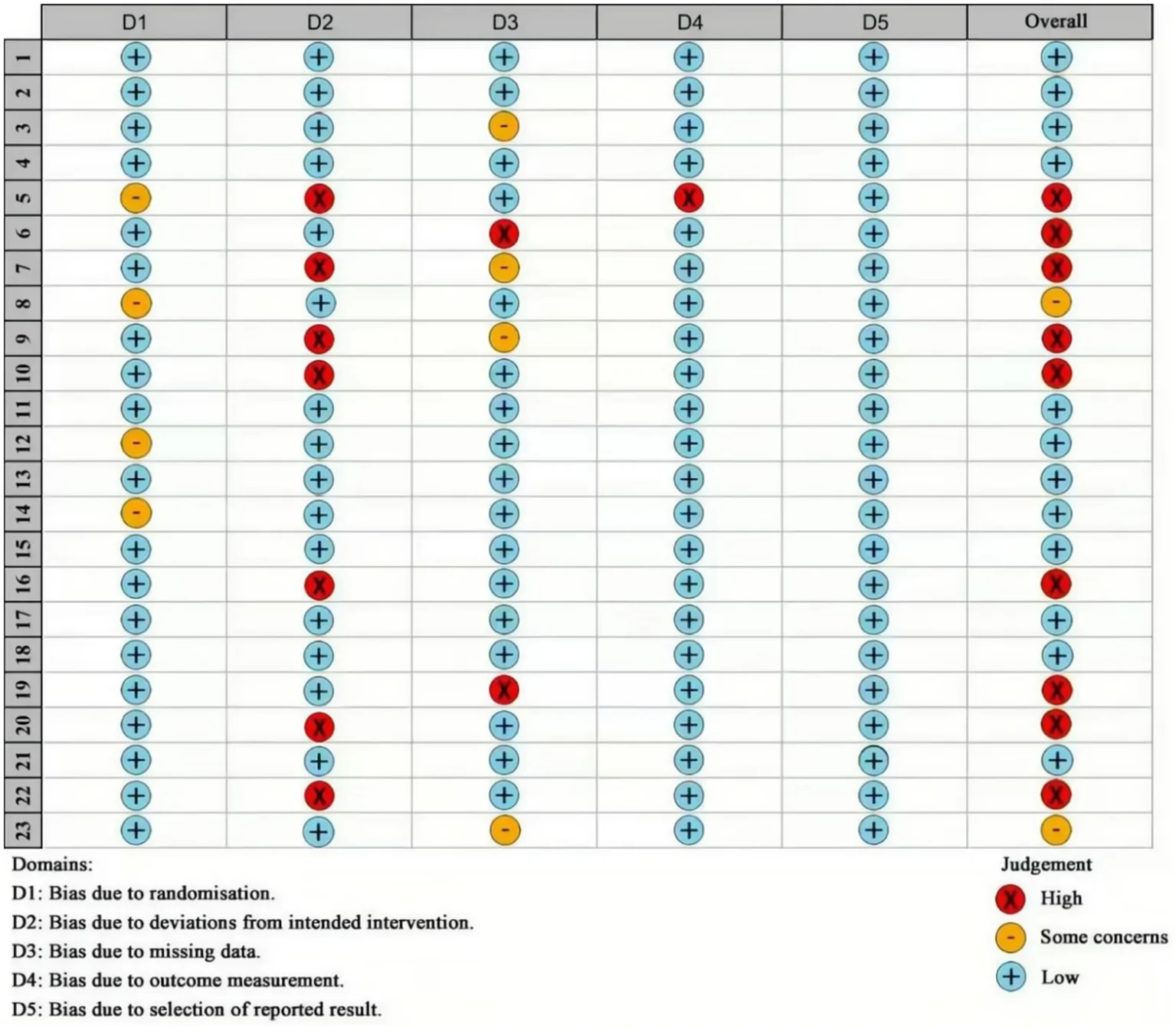
Risk of bias assessment of included studies.

**Figure 3:**
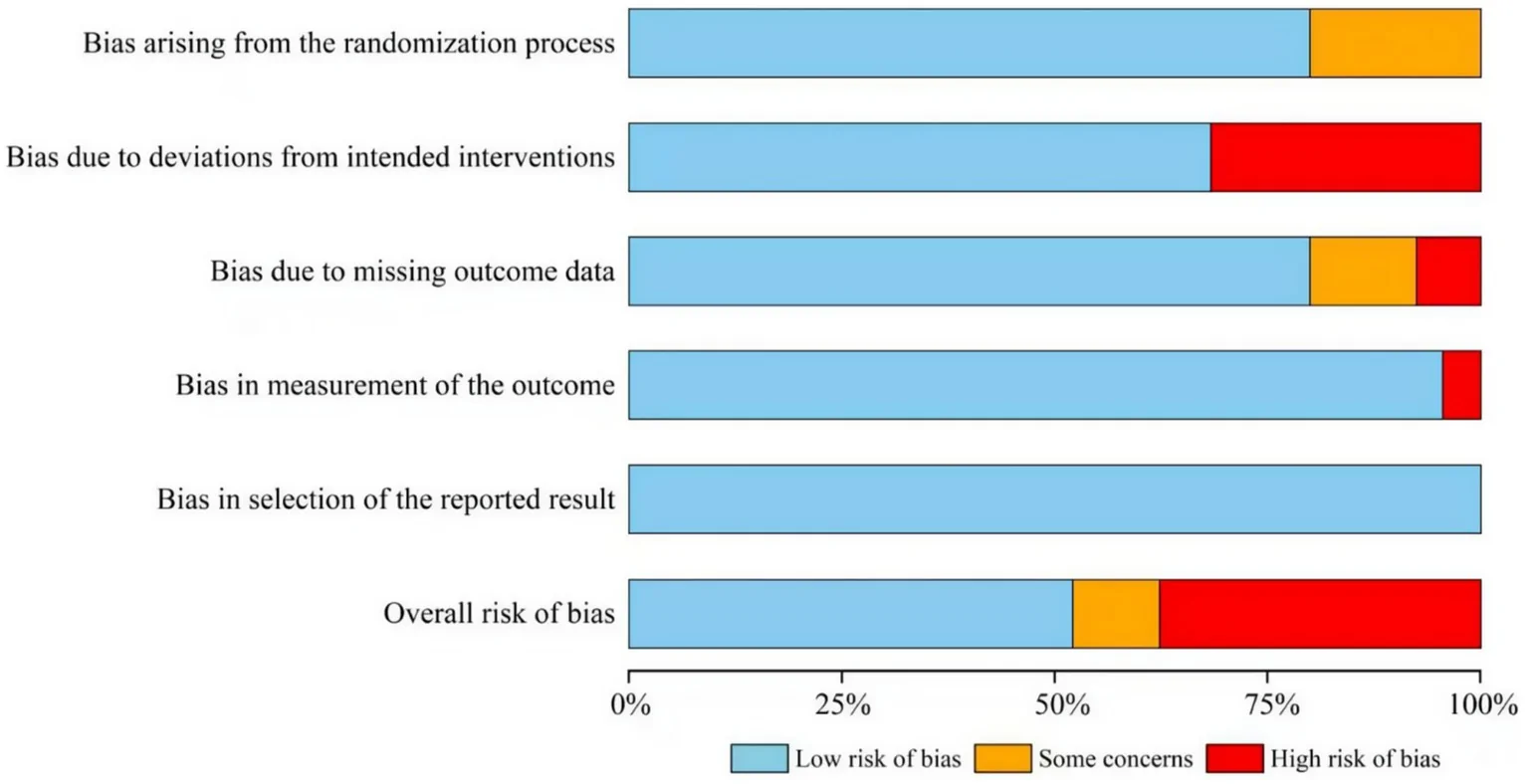
Risk of bias of included studies.

The detailed risk of bias assessment results are presented in Figures 2, 3, which document the ratings and classifications for each study according to the various risk of bias criteria.

### 3.4 Network meta-analysis

**Figure 4:**
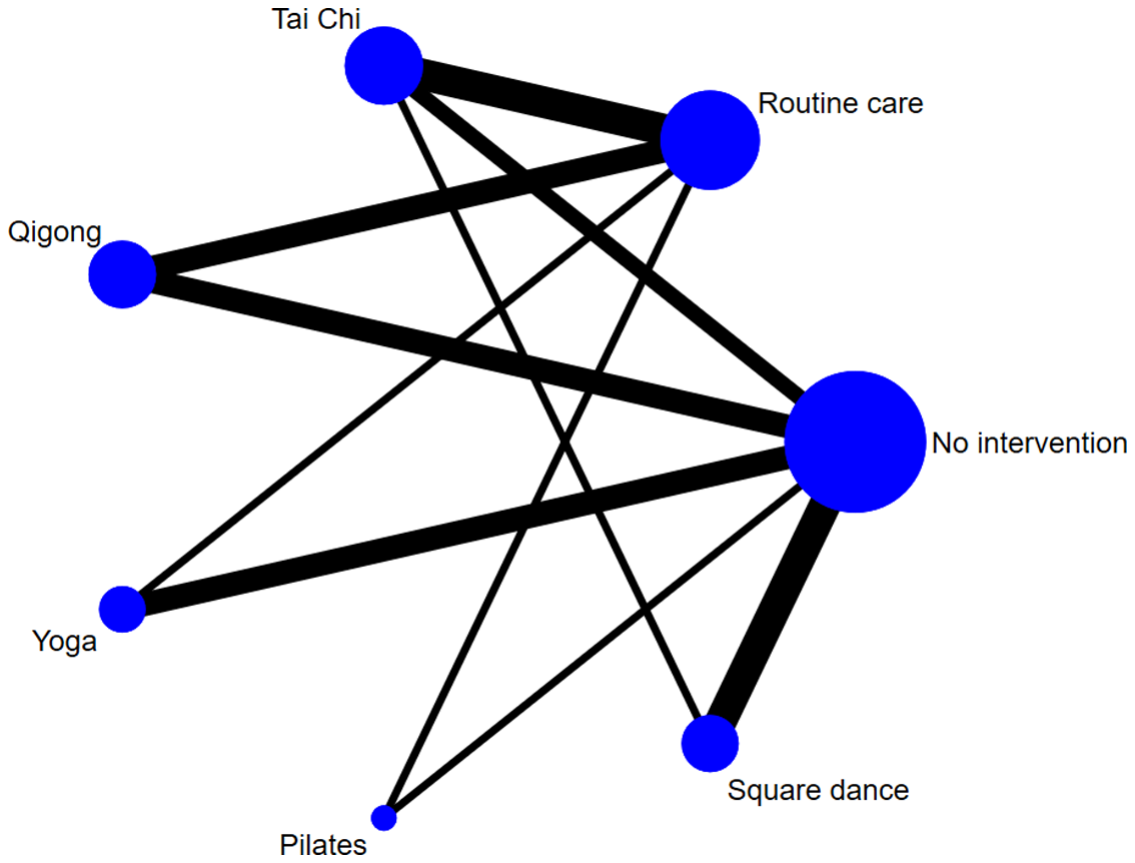
Network diagram. Each node represents an intervention or control condition. Node size is proportional to the total sample size, and line thickness is proportional to the number of direct RCT comparisons. RC, routine care; NI, no intervention.

Figure 4 presents the evidence network plot of our network meta-analysis, visualizing direct comparisons between all included mind–body exercise interventions and control conditions. Nodes represent interventions (Tai Chi, Pilates, Yoga, Qigong, Square Dancing) and controls (Routine care, No intervention), with size proportional to total RCT sample size; connecting lines denote direct pairwise comparisons, with thickness reflecting the number of supporting RCTs. Tai Chi, Qigong and Square Dancing had the largest sample sizes among active interventions, while No intervention was the largest control group. The thickest line linking Tai Chi and Routine care indicates this was the most frequently studied comparison. Node-splitting tests detected no global inconsistency across the whole evidence network, yet significant local inconsistency (*p* < 0.05) existed in pairwise comparisons involving Tai Chi and Pilates (Appendix C). Obvious divergence was found between direct and indirect effect estimates for these two top-ranked interventions, which limits the reliability of pooled network estimates for these interventions. Leave-one-out sensitivity analysis further demonstrated extreme instability of Pilates’ ranking and effect estimates, revealing high sensitivity to the inclusion or exclusion of each of its two constituent trials; minor changes to the Pilates evidence base lead to substantial shifts in its relative efficacy position and statistical credibility (Appendix D). Sensitivity analyses further confirmed the stability of our findings for Tai Chi, Yoga, Qigong and Square Dancing (Appendix D).

**Figure 5:**
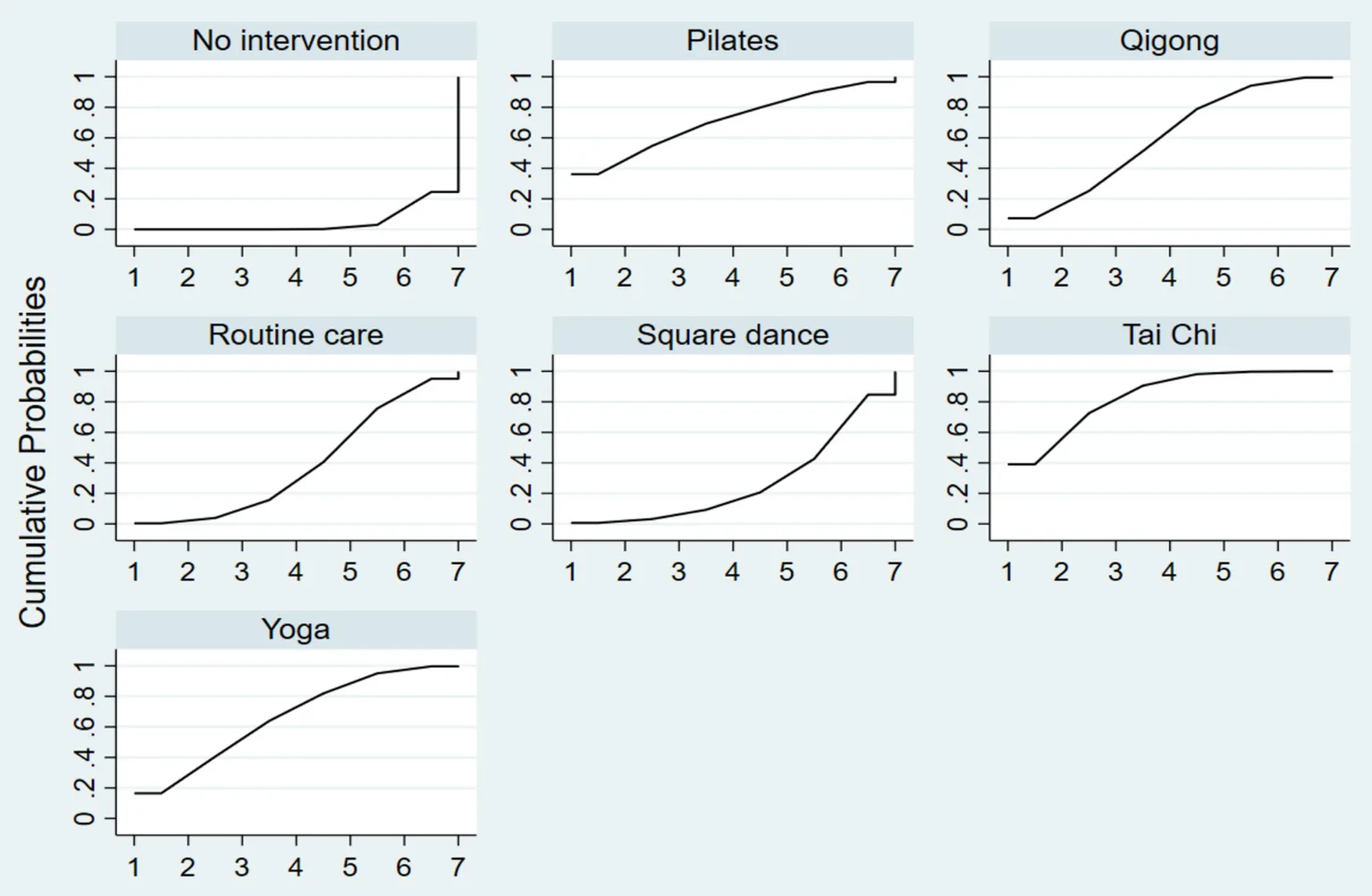
Forest plot of network meta-analysis estimates. SMDs with 95% CIs summarize direct and indirect evidence. Negative values indicate greater reductions in depressive symptoms according to the comparison direction; 95% CIs crossing zero indicate statistical uncertainty.

The forest plot (Figure 5) presents SMDs and 95% CIs from the network model, incorporating both direct and indirect evidence. Negative SMDs indicate greater reductions in depressive symptoms for the intervention listed as the comparator according to the direction specified in the league table. Because several 95% CIs were wide and crossed zero, the plot should be interpreted as showing estimated trends and uncertainty rather than definitive superiority for all active comparisons.

The following results directly compare the intervention effects of different mind–body exercises (expressed as SMD with 95% CIs). Although Tai Chi ranked favorably in SUCRA analysis, several active-intervention comparisons were not statistically significant because their 95% CIs included the null value. For example, Tai Chi compared to Pilates [SMD = −0.51 (95% CI: −7.02, 6.00)], Yoga [SMD = −1.22 (95% CI: −5.87, 3.42)], and Qigong [SMD = −1.81 (95% CI: −5.84, 2.22)]; the improvement advantage of Tai Chi is particularly pronounced when compared to Square Dancing [SMD = −4.14 (95% CI: −8.40, 0.12)]. The intervention effect of Pilates ranks second after Tai Chi, showing more favorable outcomes compared to Yoga [SMD = −0.71 (95% CI: −7.44, 6.02)], Qigong [SMD = −1.30 (95% CI: −7.83, 5.24)], and Square Dancing [SMD = −3.63 (95% CI: −10.48, 3.23)]. Yoga’s intervention effects are superior to those of Qigong and Square Dancing, with slight advantages over Qigong [SMD = −1.81 (95% CI: −6.21, 2.59)] and Square Dancing [SMD = −2.92 (95% CI: −7.71, 1.87)]. The effectiveness of Qigong is comparable to that of Square Dancing, showing no significant difference when compared to Square Dancing [SMD = −2.33 (95% CI: −6.90, 2.23)], but demonstrating better improvement effects compared to no intervention [SMD = −4.00 (95% CI: −7.32, −0.68)]. Detailed results can be found in Table 2.

**Table 2:** League table on interventions.

Tai Chi	Pilates	Yoga	Qigong	Routine care	Square dance	No intervention
Tai Chi	−5.30 (−11.20,0.60)	−4.59 (−8.05,-1.13)	−4.00 (−7.32,-0.68)	−2.78 (−6.29,0.74)	−1.67 (−5.09,1.75)	1.67 (−1.75,5.09)
−5.81 (−9.38,-2.24)	Pilates	−2.92 (−7.71,1.87)	−2.33 (−6.90,2.23)	−1.11 (−5.58,3.37)	3.63 (−3.23,10.48)	5.30 (−0.60,11.20)
−4.14 (−8.40,0.12)	−3.63 (−10.48,3.23)	Yoga	1.30 (−5.24,7.83)	2.52 (−3.91,8.95)	2.92 (−1.87,7.71)	4.59 (1.13,8.05)
−0.51 (−7.02,6.00)	−0.71 (−7.44,6.02)	0.71 (−6.02,7.44)	Qigong	1.81 (−2.59,6.21)	2.33 (−2.23,6.90)	4.00 (0.68,7.32)
−1.22 (−5.87,3.42)	−1.30 (−7.83,5.24)	−0.59 (−5.12,3.95)	0.59 (−3.95,5.12)	Routine care	4.14 (−0.12,8.40)	5.81 (2.24,9.38)
−1.81 (−5.84,2.22)	0.51 (−6.00,7.02)	1.22 (−3.42,5.87)	1.81 (−2.22,5.84)	3.03 (−0.12,6.19)	Square dance	2.78 (−0.74,6.29)
−3.03 (−6.19,0.12)	−2.52 (−8.95,3.91)	−1.81 (−6.21,2.59)	−1.22 (−4.59,2.14)	1.22 (−2.14,4.59)	1.11 (−3.37,5.58)	No intervention

Bold dark-colored values indicate statistically significant results.

**Figure 6:**
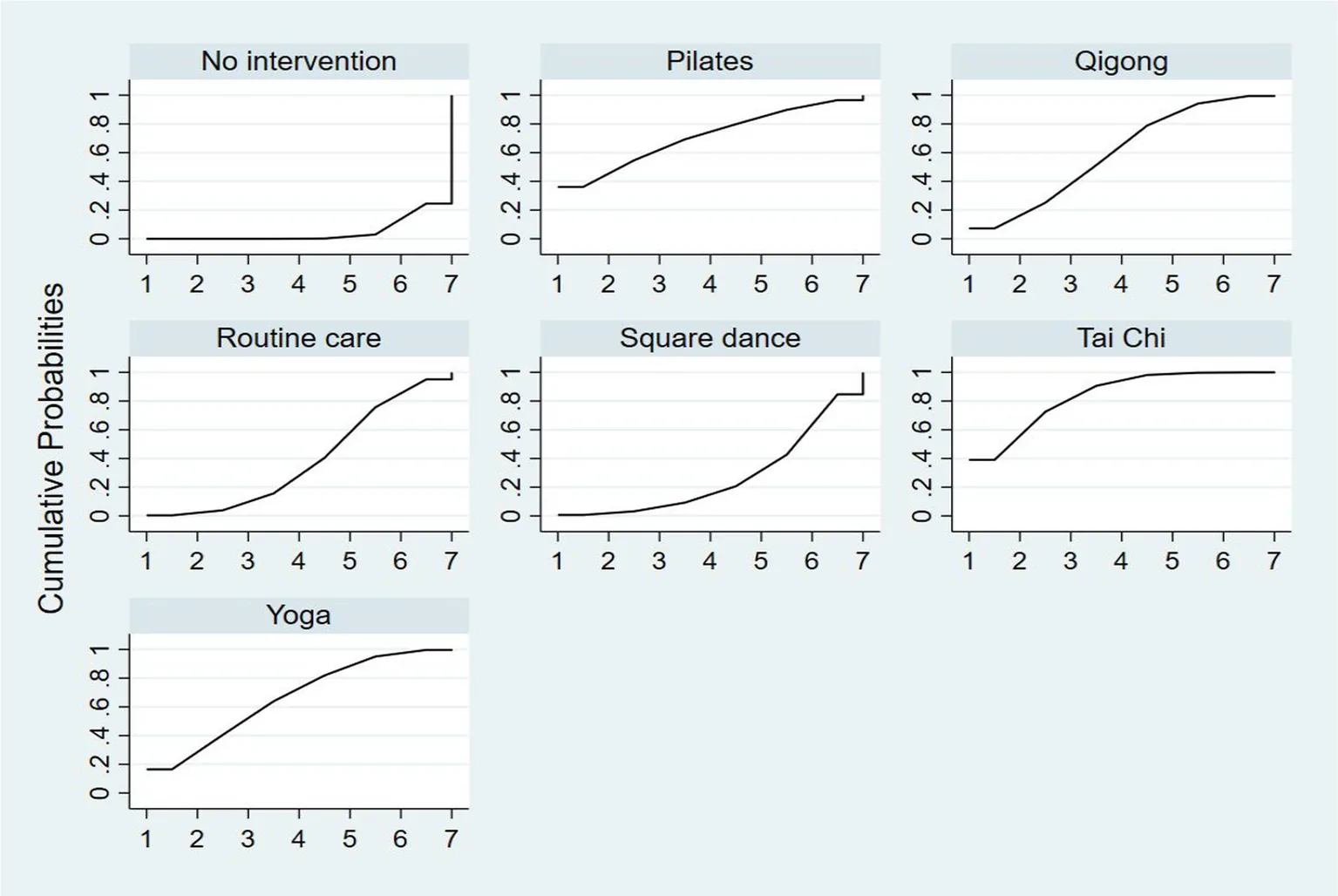
SUCRA ranking plot. SUCRA values represent the probability that each intervention ranks among the more beneficial treatments. Higher values indicate higher ranking probability but should not be interpreted as definitive superiority when pairwise CIs are wide or cross zero.

These SMD values quantify the differences in the efficacy of various mind–body exercises in improving depression among older adults, where smaller absolute values reflect stronger effects. The SUCRA rankings were used to estimate the relative efficacy probability of each intervention, with the caveat that the evidence network was unbalanced due to varying sample sizes of included studies for different mind–body exercises. Tai Chi showed the highest efficacy probability (SUCRA = 83.3%), supported by seven included RCTs with relatively sufficient sample size. Pilates had the second-highest SUCRA value (71.0%) and a 36.1% probability of being the “best treatment”; however, this result was supported by only two RCTs and therefore should be interpreted cautiously. Yoga and Qigong followed with SUCRA values of 66.3 and 59.4%, respectively. The probabilities of being the “best treatment” for Tai Chi (39.0%), Pilates (36.1%), and Yoga (16.5%) together accounted for 91.6% of the total probability (Table 3; Figure 6). GRADE-NMA evidence quality assessment results are provided in Appendix E.

**Table 3:** Ranking of SUCRA probabilities.

Intervention Name	SUCRA (%)	Probability of best (%)	Rank
Tai Chi	83.3	39.0	1
Pilates	71.0	36.1	2
Yoga	66.3	16.5	3
Qigong	59.4	7.3	4
Routine care	38.5	0.4	5
Square dance	26.8	0.7	6
No intervention	4.6	0.0	7

Dark shades indicate the top three ranked interventions.

### 3.5 Publication bias

**Figure 7:**
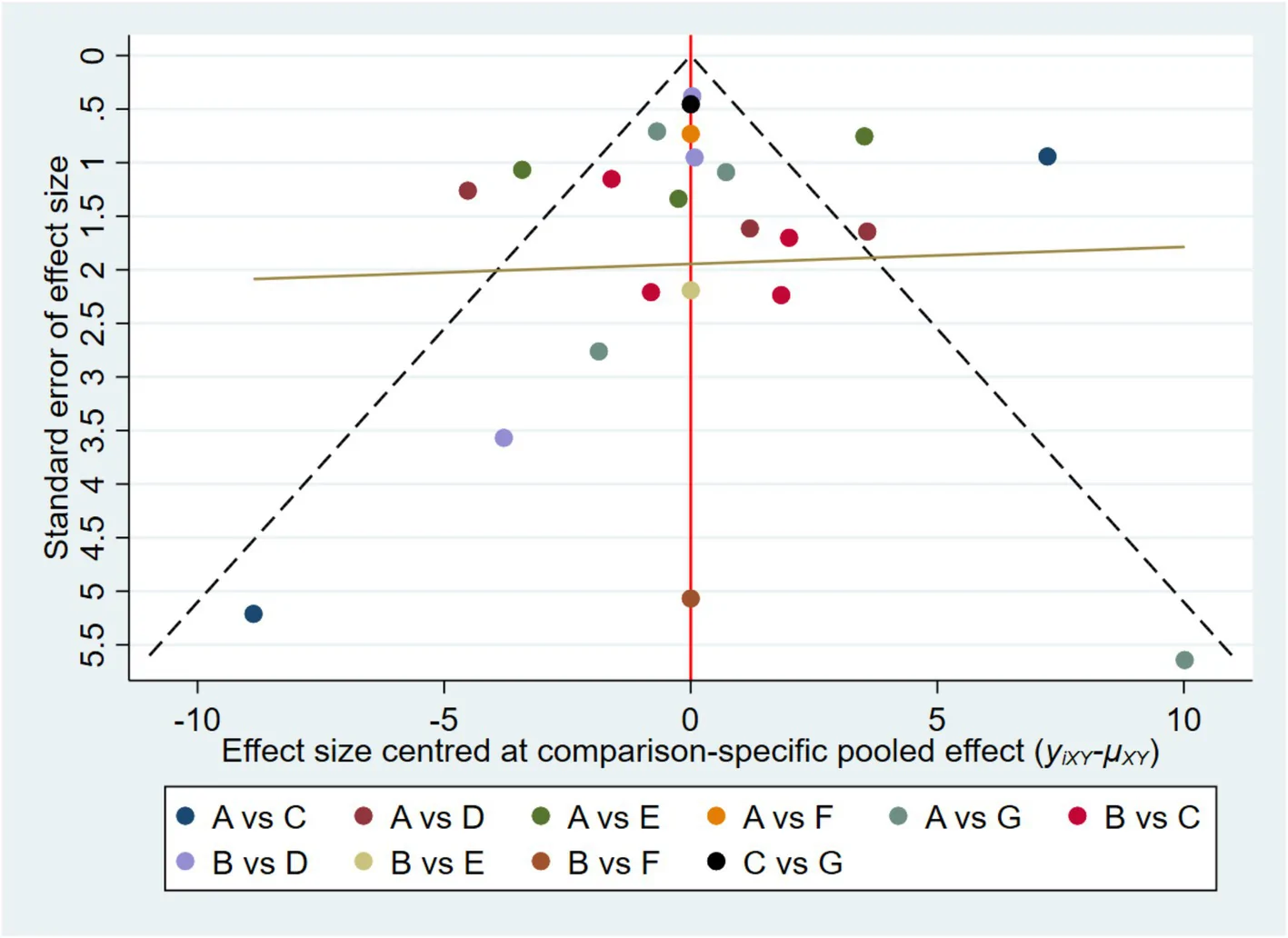
Funnel plot on publication bias.

As illustrated in Figure 7, this study initially employed a funnel plot to assess publication bias. The distribution of included studies in the funnel plot appears to be generally symmetrical, and visual inspection did not reveal any significant signals of publication bias, suggesting that the potential publication bias in this study was mild and its impact on the overall effect size was limited. However, it should be acknowledged that publication bias is inherently unavoidable in meta-analyses, as studies with statistically significant or positive intervention effects are more likely to be published than those with negative or null results, which may lead to a slight overestimation of the efficacy of mind–body exercises in alleviating depressive symptoms in older adults. Publication bias was assessed using Begg’s rank correlation and Egger’s linear regression tests, with detailed results presented in Table 4. Begg’s test revealed no significant publication bias (z = −0.2377, *p* = 0.812), and Egger’s test similarly showed no evidence of meaningful bias (bias term = 0.2999, *t* = 0.2999, *p* = 0.767). These consistent findings confirm that selective reporting is unlikely to have substantially affected the pooled effect estimates.

**Table 4:** Publication bias: Begg’s and Egger’s tests.

Test	Statistic	Value	Std. Err.	*t*-value	*p*-value	95%CI
Begg’s test	Kendall’s Score (P-Q)	−9.0000	–	–	–	–
	Std. Dev. of score	37.8638	–	–	–	–
	*z*-value	−0.2377	–	–	0.812	–
	*z*-value (continuity corrected)	−0.2113	–	–	0.833	–
Egger’s test	Slope bias	1.5403	1.0346	1.4888	0.151	−0.6113, 3.6918
	Intercept	0.2956	0.9858	0.2999	0.767	−1.7546, 2.3458

## 4 Discussion

This network meta-analysis evaluates the effects of various mind–body exercise interventions on alleviating depressive symptoms in older adults. Based on a comprehensive analysis of 23 randomized controlled trials, our findings indicate that Tai Chi and Pilates show the most promising non-pharmacological effects for reducing depression in this population. Notably, Tai Chi emerged as the intervention with the highest SUCRA probability (83.3%), closely followed by Pilates (SUCRA = 71.0%). However, these rankings should be interpreted cautiously because several comparisons between active interventions were not statistically significant and some confidence intervals were wide. Therefore, the findings support Tai Chi as a promising option rather than definitive evidence of its superiority over all other mind–body exercises.

Depression in older adults is a persistent emotional disorder triggered by the natural decline of physiological functions and the interaction of multiple factors. It primarily stems from physiological changes such as reduced neurotransmitter secretion, decreased hormone levels, and the accumulation of chronic illnesses, which interact with psychosocial stressors such as bereavement, empty nest syndrome, and role regression (1, 2). Neurotransmitter imbalances (e.g., decreased serotonin and dopamine levels) directly disrupt emotional regulation pathways, while physical pain and social withdrawal associated with chronic diseases exacerbate negative emotions, creating a vicious cycle of “physiological damage and psychological repression” that significantly heightens the risk of depression and increases the likelihood of chronic symptoms in older adults (3, 4). Mind–body exercises, characterized by low intensity and gentle rhythm (25), promote neurotransmitter secretion, inhibit inflammation, alleviate physical pain, and specifically target the physiological foundations of disease (26). They also facilitate emotional regulation, reduce negative thinking, and enhance self-worth through coordinated practice, thereby alleviating psychological repression. Furthermore, their adaptable nature (supporting both group participation and independent practice for older adults living alone) allows for convenient implementation without the need for specialized equipment or venues, fostering social connections, promoting mindfulness and acceptance, and strengthening social support networks. This makes them a sustainable threefold intervention that addresses the complex physiopsychosocial mechanisms underlying depression in older adults (27), thus meeting the practical needs of this demographic and providing a safe and effective non-pharmacological intervention option.

Tai Chi, characterized by the integration of movement and stillness, along with synchronized breathing and intention, may improve depressive symptoms in older adults through several plausible pathways (31, 32, 48). Physiologically, the smooth and coherent movements of Tai Chi foster a beneficial interaction with breath regulation, which promotes the synthesis and release of mood-regulating neurotransmitters like serotonin and dopamine, directly repairing the imbalanced pathways central to depression and alleviating symptoms such as low mood and loss of interest (54). Additionally, Tai Chi inhibits the excessive expression of inflammatory markers such as IL-6 and TNF-*α*, reducing chronic inflammation’s impact on emotional centers in the brain. It also improves hypothalamic–pituitary–adrenal (HPA) axis function and lowers cortisol levels, breaking the vicious cycle where inflammation exacerbates depression and vice versa (55, 56). However, most included RCTs in the present review assessed depressive symptoms rather than biological markers; therefore, these pathways remain inferential in the context of this network meta-analysis. From an intervention perspective, Tai Chi’s “mind–body connection” is not merely an additive effect of physical movement and psychological regulation; rather, it involves a practice model guided by intention that activates the emotional regulation functions of the prefrontal cortex, reduces hyperactivation of the amygdala, and may target the negative cognitive biases associated with depression (54). It’s simple and gentle movements do not require high physical exertion, making it suitable for older adults at risk of depression due to age, chronic illness, or mobility limitations. It supports both group and solitary practice modes, alleviating feelings of loneliness through social connections while ensuring the sustainability of the intervention. These features may explain why Tai Chi achieved the highest SUCRA ranking, but the non-significant comparisons with several active interventions indicate that Tai Chi should be regarded as a promising and feasible option rather than the unequivocally optimal intervention (57, 58).

Although Pilates showed a relatively high SUCRA ranking (second, 71.0%) in this study, this result was only supported by two included RCTs with limited sample size and thus needs to be cautiously interpreted. Its effectiveness in alleviating depressive symptoms stems from its chain mechanism of “core control, breath regulation, and self-efficacy enhancement,” which specifically addresses the pathological characteristics of depression in older adults (42, 45, 59). Physiologically, Pilates focuses on strengthening core muscle groups and enhancing body control. Through precise muscle contraction and relaxation training, it improves posture stability in older adults, reducing the overlap of chronic pain, such as lower back pain, with depressive emotions, thus preventing the amplification of negative feelings (60). Simultaneously, its emphasis on synchronizing breath with movement helps regulate autonomic nervous system balance, lowering sympathetic nervous system excitability and promoting GABA secretion, which may directly alleviate anxiety and emotional fluctuations accompanying depression (61). From an intervention standpoint, Pilates’ requirement for precision significantly enhances older adults’ sense of bodily control, thereby boosting self-efficacy. This process of “gaining a sense of psychological control through manageable physical practice” effectively may help break the vicious cycle of “helplessness and negative cognition” characteristic of depression, playing a crucial role in improving symptoms such as low mood and self-denial (62, 63). However, the limited sample size of Pilates studies and insufficient standardization of intervention duration and frequency (12–16 weeks, twice weekly) restricts the stability of the observed effects on depression.

Yoga may improve depressive symptoms through dual pathways of “neuroprotection and mindfulness regulation,” although its effectiveness is constrained by adaptability issues (41, 44, 64). Physiologically, yoga’s stretching movements promote overall blood circulation, enhance cerebral blood flow and oxygenation, increase the expression of brain-derived neurotrophic factor (BDNF), protect neuronal synaptic function, and delay cognitive decline associated with depression, indirectly mitigating emotional problems arising from cognitive deterioration (65). Additionally, stretching practices can relieve muscle tension and reduce the negative impact of physical pain on emotions, preventing the overlapping effects of pain and depression (66). On the intervention level, yoga’s integrated mindfulness approach guides older adults to focus on present bodily sensations, diminishing ruminative negative thinking, and directly addressing cognitive abnormalities at the core of depression, thus aligning with the psychological intervention logic for depressive emotions (41, 46, 47). Nevertheless, the improvement effects of yoga on depression are inferior to those of Tai Chi and Pilates, as some poses require a certain level of physical flexibility and strength, making it less suitable for older adults with limited joint flexibility, resulting in low participation rates and adherence that hinder sustained improvement of depressive symptoms (67). Moreover, yoga emphasizes individual mindfulness experience and has weaker group interaction, lacking the social support dimension necessary for effectively regulating emotions.

Qigong improves depression in older adults through its core mechanisms of “regulating organ function and energy, and inflammation suppression,” yet the singularity of its intervention dimensions limits its effectiveness (39, 40, 52, 53). Physiologically, Qigong’s fluid and coherent movements, centered on guiding the flow of energy and blood through intention, can enhance organ function, regulate the neuroendocrine-immune network, and significantly lower the levels of inflammatory markers such as TNF-*α*, alleviating the pathological state of depression caused by chronic inflammation and reducing sustained stimulation of emotional centers by inflammation (68). Additionally, its calming breathing exercises can regulate HPA axis function, decrease cortisol secretion, and improve neurotransmitter imbalances, directly alleviating symptoms such as low mood and fatigue (69). However, the effectiveness of Qigong in improving depression may be limited; first, it focuses more on individual internal regulation of energy and blood, with weak social interaction attributes during group practice, making it difficult to strengthen psychological support through social connections, hence inadequately addressing the social dimension of depression in older adults (36, 37). Secondly, the “intentional guidance” aspect of Qigong poses comprehension and execution difficulties for some older adults with mild cognitive decline, limiting its adaptability and resulting in a narrow coverage for depression improvement (39, 53).

Compared to the first four types of mind–body exercises, square dancing appears to show significantly weaker effects on alleviating depression, primarily due to its lack of targeted physiological interventions and superficial psychological adjustments that fail to accurately address the core pathogenic mechanisms of depression in older adults (43, 48). From a physiological perspective, square dancing is characterized by collective participation and a strong rhythmic pattern. Although it qualifies as aerobic exercise and can somewhat promote neurotransmitter secretion, it lacks specific regulatory mechanisms targeting the core physiological abnormalities of depression (such as inflammatory markers and HPA axis function), preventing it from fundamentally improving the physiological basis of depression (49, 50). Additionally, its strong rhythmicity may create psychological pressure for some emotionally sensitive depressed older adults, potentially activating sympathetic nervous system excitation and exacerbating emotional fluctuations, which is counterproductive to alleviating depression (70). Regarding intervention mechanisms, the primary advantage of square dancing lies in its social connections—group practice can alleviate feelings of loneliness and enhance social support, which is somewhat helpful for improving mild depression due to loneliness. However, this advantage does not compensate for its key shortcomings: first, the high repetition of movements lacks the core design of “mind–body connection,” preventing direct adjustment of emotional cognition and negative thinking at the core of depression, rendering the psychological intervention dimension superficial (71); second, it relies heavily on group settings and music coordination, making it poorly suited for older adults living alone or those with mobility issues, presenting significant implementation challenges (72); third, the long-term repetition of movements can lead to burnout, insufficient intervention sustainability, and some high-intensity steps (e.g., rapid turns, jumps) might pose safety risks for older adults, further limiting the applicability of depression improvement (50). These factors lead to square dancing having a social support advantage while failing to form effective interventions targeting the core physiopsychological mechanisms of depression in older adults, only showing some improvement in mild loneliness-related depression, with overall effects being slightly better than routine care and no intervention.

In summary, this study, through network meta-analysis, provides an indication of the relative intervention effects of various mind–body exercises on depressive symptoms in cognitively intact older adults without other physical illnesses based on the existing evidence base. Tai Chi showed the most promising efficacy among the tested interventions and can be considered a preferred reference non-pharmacological intervention option, combined with individual physical condition and adaptability.

Pilates, Yoga and Qigong can be flexibly selected as supplementary intervention measures according to individual strength, flexibility and cognitive status, with their clinical application needing to be supported by more standardized RCTs; Square Dancing showed weaker effects than routine care and other mind–body exercises, and may only provide limited benefits for low-risk individuals with mild loneliness-related depressive symptoms as an adjunct social activity. It is crucial to note that all the above recommendations are based on the synthesis of limited existing evidence, and are not definitive clinical guidelines due to the limitations of study quantity, sample size and heterogeneity. Additionally, 39% of the included studies were rated as having a high overall risk of bias, which further tempers confidence in the SUCRA rankings, particularly for interventions such as Pilates that rely on only a few trials.

## 5 Strengths and limitations

This study presents several notable strengths. First, it is the first network meta-analysis, to our knowledge, specifically targeting older adults without cognitive impairment or other physical/mental disorders, thereby addressing a critical evidence gap and providing tailored data for non-pharmacological depression interventions in this population. Second, the methodology strictly adheres to the PRISMA 2020 guidelines, with explicit eligibility criteria, inclusion of only high-quality RCTs, and independent dual screening and data extraction (Cohen’s *κ* = 0.73–0.81), which collectively enhance the robustness of the findings. Third, the analytical framework incorporates multiple quality-assurance procedures, including funnel plots for publication bias, node-splitting tests for consistency, random-effects models for heterogeneity, and systematic extraction of core intervention parameters (duration, frequency, intensity), further improving the reliability and comparability of the results.

Several methodological limitations warrant consideration. First, the content of control conditions—especially “routine care”—varied considerably across trials, often lacking standardization or explicit definition (e.g., health education, usual follow-up, or no structured activity). Such heterogeneity may introduce confounding in indirect comparisons and reduce the precision of effect estimates relative to a truly uniform comparator. Moreover, inadequate reporting of concurrent treatments in some trials makes it difficult to attribute observed effects solely to mind–body exercises rather than to differential background care. Thus, network estimates comparing active interventions with routine care should be interpreted cautiously. Second, the distribution of studies across exercise types is highly uneven. Pilates is only supported by two RCTs, and leave-one-out sensitivity analysis confirmed its ranking and pooled effect estimates exhibit extreme instability and high sensitivity to single-study removal. The small evidence base renders all inferences for Pilates highly contingent on individual trial data, severely weakening the credibility of its second-place SUCRA ranking. Yoga and Qigong protocols also show wide variation in duration (8–24 weeks) and frequency (1–5 sessions/week), precluding any firm conclusion about optimal dosage. Third, node-splitting tests revealed significant local inconsistency in pairwise comparisons involving Tai Chi and Pilates, with inconsistent direct and indirect effect estimates for these two top-ranked interventions, which impairs the reliability of their relative efficacy rankings derived from the network meta-analysis. Other limitations include: potential language bias from restricting to English-language publications; lack of subgroup analyses by gender, depression severity, or baseline health, which limits generalizability to specific older adult subgroups; and the absence of long-term follow-up, preventing assessment of symptom sustainability. Finally, although activities like Square Dancing have limited physiological and psychological targeting, no optimization strategies were examined, constraining practical applicability.

Future research should prioritize expanding the evidence base for under-represented modalities such as Pilates and Yoga through larger, well-powered RCTs with standardized protocols (e.g., specified intensity, frequency, and duration). Established tai chi frameworks could serve as references for defining appropriate intensity thresholds (e.g., %HRmax, METs) to inform clinical prescription. Inclusion of non-English studies would mitigate language bias, while incorporation of moderating variables (gender, depression severity, comorbidities) would facilitate personalized intervention strategies. Long-term follow-up is essential to evaluate the durability of antidepressant effects across the prevention–intervention–maintenance continuum. For less effective modalities like Square Dancing, integrating mindfulness components or low-intensity movement designs may enhance their therapeutic targeting and broaden their clinical utility.

## 6 Conclusion

This study synthesizes the existing RCT evidence to compare the effects of different mind–body exercises on depressive symptoms in older adults without cognitive impairment and other physical illnesses. The findings indicate that Tai Chi exhibits the highest efficacy probability (SUCRA = 83.3%), followed by Pilates (SUCRA = 71.0%). However, two critical limitations weaken confidence in this ranking: first, Pilates evidence is limited to merely two RCTs, and leave-one-out sensitivity analysis proves its efficacy ranking and effect estimates are extremely unstable and highly sensitive to single-trial exclusion; second, node-splitting tests identified significant local inconsistency in pairwise comparisons of Tai Chi and Pilates, showing clear divergence between direct and indirect evidence. Yoga (SUCRA = 66.3%) and Qigong (SUCRA = 59.4%) also demonstrated clinically observable improvements, whereas Square Dancing yielded relatively weaker effects.

Based on the current limited evidence, Tai Chi can only be regarded as a tentative reference non-pharmacological intervention option for this older adult population in clinical practice. Pilates, Yoga or Qigong can be flexibly selected according to the individual age, physical function (e.g., strength, flexibility) and social needs of older adults; yet Pilates cannot be confidently recommended due to the extreme sensitivity of its results to small changes in its limited trial sample; Square Dancing might be considered as an auxiliary social intervention measure for low-risk individuals with mild loneliness-related depressive symptoms. Moreover, with 39% of the included RCTs carrying a high overall risk of bias, alongside local inconsistency in the network estimates of the two highest-ranked interventions, the reliability of these efficacy rankings is further constrained. Due to uneven study distribution, small sample sizes for partial interventions, heterogeneous intervention protocols and local inconsistency within the evidence network, all the above recommendations need to be verified by more high-quality RCTs, and cannot be regarded as definitive clinical first-line intervention guidelines.

Standardization of intervention parameters (such as frequency, intensity, and duration) for various mind–body exercises is an urgent need to improve the clinical reproducibility and evidence robustness of mind–body exercise interventions. Future research should focus on recruiting larger sample sizes and conducting more standardized Pilates trials to resolve the high sensitivity and instability of current effect estimates, expand high-quality trials for Tai Chi to resolve the observed local inconsistency, developing standardized, personalized exercise plans based on the age, baseline health status and depression severity of older adults, conducting long-term follow-up studies to verify the sustained intervention effects of mind–body exercises, and carrying out targeted mechanistic research combined with physiological and psychological indicators to provide a more solid evidence base for the clinical application of mind–body exercises in alleviating depressive symptoms in older adults.

## Data Availability

The original contributions presented in the study are included in the article/Supplementary material, further inquiries can be directed to the corresponding author/s.
